# A feasibility study of individual 3D-printed navigation template for the deep external fixator pin position on the iliac crest

**DOI:** 10.1186/s12891-020-03509-6

**Published:** 2020-07-21

**Authors:** Bin Liang, Qiang Chen, Shuai Liu, Shuo Chen, Qingqiang Yao, Bo Wei, Yan Xu, Cheng Tang, Liming Wang

**Affiliations:** 1 Department of Orthopaedic Surgery, Nanjing First Hospital, Nanjing Medical University, Nanjing, 210006 China; 2 School of Biological Science & Medical Engineering, Southeast University, Nanjing, China; 3 Digital Medicine Institute, Nanjing Medical University, Nanjing, 210006 China; 4 Department of Orthopaedic Surgery, Xuzhou Central Hospital, Xuzhou, 221000 China; 5 Engineering Research Center in Biomaterials, Sichuan University, Chengdu, 610065 China

**Keywords:** Individual, Template, Pelvis fracture, External fixators

## Abstract

**Background:**

The aim of this study was to investigate the feasibility of an individual navigation template for the deep pin position on the iliac crest, based on digital design and 3D printing technology.

**Methods:**

The preoperative CT images of 8 patients with pelvic fractures were collected. The data were reconstructed using a 3D imaging reconstruction workstation. An individual navigation template for the deep pin position on the iliac crest was designed on a virtual 3D model. The individual drill template and the solid pelvic model were produced using the 3D printing technology. The individual drill template was used for intraoperative deep pin position on the iliac crest after the preoperative simulation was completed.

**Results:**

Thirty-two external fixator pins were inserted using the individual drill template. The average depth of pins was 84.82 mm. The trajectories were appropriate based on the postoperative X-ray and CT scan. No significant difference in the entry point, convergence angle, and caudal angle of the pins were noted before and after the operation (all *P* > 0.05). Finite element analysis indicated that the deep external fixator pin position could more reasonably distribute the stress in the cortical and spongy bones in the pelvis. All patients could perform partial weight-bearing activity 6 weeks postoperatively. No loosening and rupture of the pin, infection, and no damage of blood vessels and nervous tissue were found during the entire follow-up period.

**Conclusions:**

The individual drill template technique is an improvement of the traditional technique, which could increase precision and the depth of pin position. In addition, good mechanical stability and low risk of pin-related complications occurred due to the individual drill template, which makes the external fixation technique a potential alternative.

## Background

Pelvic fractures are usually caused by high-energy trauma, such as traffic accident or falls from heights in young population, and with a consequent of associated injuries. In the elderly population with osteoporosis, fragility fractures can often be caused by low-energy trauma, such as a fall from standing or some minor trauma. The overall mortality rate worldwide varies from 6 to 35% [1, 2] . External fixation plays an important role in the reduction of pelvic volume, pain, and bleeding for the high-energy trauma and the fragility fracture. The traditional external fixation on the iliac crest is the commonly used surgical technique because it is minimally invasive and easy to perform [3]. However, it is difficult to control the direction of external fixator pins using the traditional surgical technique, which often results in the pin tip easily penetrating the cortex of the ilium during the surgery. In contrast with our experience using the percutaneous approach, correct pin placement using the anterosuperior approach is particularly difficult for inexperienced surgeons, Wailakul et al. reported that the incidence rate of incorrect pin placement up to 18% at the iliac crest for external fixation [4]. In addition, the incorrect pins are prone to infection and loosening, which greatly affects the mechanical stability of external fixation [5]. It is reported that the strong pin purchase can be achieved due to the high bone density in the supraacetabular region of human pelvis [6]. We wanted to investigate a simple modification of the traditional method to place pin from the iliac crest toward the supraacetabular region, thus to improve the mechanical stability of external fixation. To the best of our knowledge, there are no reported studies improving the current surgical technique until now [7].

Rapid prototyping (RP) is an emerging industrial technique. Its application combined with reverse engineering technology in the field of medicine has made precise and individualized treatment possible. The technique is especially important in case of minimally invasive surgeries. Preoperative design and surgical planning, along with the application of a 3D-printed navigation template, can effectively reduce errors associated with lack of experience and poor operative techniques. Currently, navigation templates are used to guide the surgical insertion of internal fixation screws and plates. However, its application in the deep pin positioning on the iliac crest has not been reported. The aim of this study was to use digital 3D reconstruction, reverse modeling technology, and 3D print to produce an individual navigation template. The template was designed to serve as a guide to increase the precision and depth of external fixator pin positioning on the iliac crest.

## Methods

### General information

This research was authorized by Nanjing First Hospital’s ethics committee. Eight pelvic-fracture patients refused expectant treatment and selected minimally invasive surgery. They were given detailed information about the study and their situation and signed the informed consent forms. The 3D navigation template was used as part of the surgical procedure for all patients. The general clinical data of the patients are listed in Table 1.

**Table 1:** The baseline characteristics of patients

No.	Age	Gender	BMI	Location of injury	Cause of injury	AO/OTA	Time from trauma to surgery
1	61	Female	23.5	Both sides	Car accident	B1	6 days
2	65	Female	29.1	Right side	Car accident	B1	6 days
3	29	Male	26.7	Right side	Fall	C1	7 days
4	42	Male	25.8	Left side	Car accident	B2	4 days
5	22	Female	28.4	Left side	Car accident	B2	1 days
6	61	Male	26.3	Left side	Car accident	B1	3 days
7	80	Male	31.2	Both sides	Car accident	C1	3 days
8	58	Male	24.9	Right side	Fall	C1	2 days

### Preoperative measurement, design, and template preparation

**Fig. 1:**
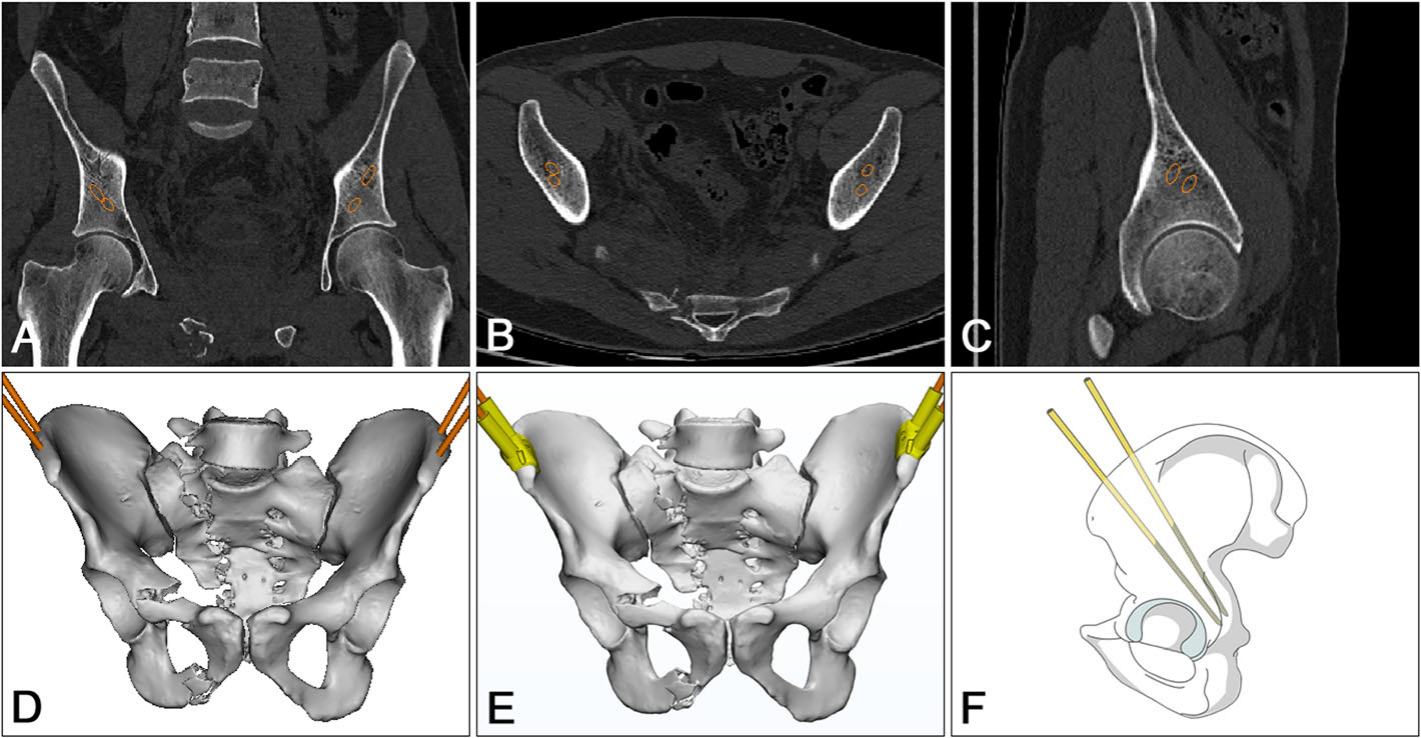
Preoperative design and template preparation. **a**–**d** DICOM data were imported into Mimics software for 3D reconstruction, and external fixator pin insertion channels were simulated. **e** The navigation template was designed with reverse modeling. **f** Ideal position of external fixator pins

All experimental methods described in this study were approved by Nanjing First Hospital and performed in accordance with the relevant guidelines and regulations. A spiral CT scan of pelvis was obtained using the following specific scan parameters: 120 KV, 120 mAs, pixel matrix 512 × 512, 1 mm thick slices, 0.5 mm interlamellar spacing. Original DICOM data were imported into Mimics software (Materialise Inc., Belgium) to produce a 3D reconstruction of pelvis. Optimal pin position in simulation module is facilitated by searching a position with adequate depth and avoiding bone penetration in the 3D reconstruction of pelvis. These data were imported into the 3D reconstruction program (STL format) in Geomagic Design Direct software to redefine the coordinate axis. Then, the trajectories of the external fixator pin were determined [8]. A navigation template with reverse modeling was designed, and the navigation template 3D model was completed with the insertion channel for the pin (Fig. 1). The template and pelvis model were produced by the fused deposition modeling (FDM) method of RP technology.

Following the preparation of the template and pelvis model, the simulated operation was performed. After matching navigation templates to the pelvic models, the external fixator pins were inserted through the insertion channel. The position and orientation of the pin were evaluated according to the preoperative plan. The templates were sterilized with low-temperature plasma before operation.

### Operation and postoperative treatment

Using a cambered incision along the iliac crest, the soft tissue was carefully separated to reveal the base of the iliac crest and the inner plate of the ilium. The 3D navigation template was used to guide the insertion of the pin as described above. After all pins have been inserted, a low-profile and anterior frame was then constructed and angled caudally. The pin-bar clamps were attached to the pins, then short connecting rods were fastened to the pins. Long rods were attached to the short connecting rods, then connected by bar-bar clamps. Double stacking the connecting rods were applied to increase frame stability.

According to the preoperative and postoperative 3D reconstructions, three parameters (entry point, convergence angle, and caudal angle) of all pins were measured and compared before and after the operation, respectively, to evaluate whether the trajectories were precise and consistent with the preoperative plans. The entry point refers to the distance from the entry point to the anterior superior iliac spine. The convergence angle refers to the axis of the pin to the sagittal plane of the pelvis. The caudal angle refers to the axis of the pin relative to the plane, which is the axis of the true conjugate (from sacral promontory to symphysis) vertical to the sagittal plane.

Postoperatively, the patients remained touch-down for 6 weeks, but there were no restrictions regarding hip motion. At 6 weeks, the patients were assessed for fracture consolidation according to regional symptoms and signs, as well as X-ray findings which included callus formation, fracture displacement and implant position. Then the patients were generally allowed to be partial weight bearing. After 12 weeks, when the fracture had mostly healed, the external fixator pins were removed and thereafter advanced to the full weight bearing.

### The finite element model and mechanical analysis

To analyze the effect of different insertion depths of the external fixator pin on the stress distribution of pelvis, two insertion depths 30 mm and 70 mm were chosen for finite element analysis. The inserting direction of pins designed in finite element analysis was consistent with that in the surgery, as well as the distance between the entry points of the pelvis and the connectors which connected the pins and the frame. Based on the postoperative CT images (slice number: 489, slice thickness: 1 mm) of a pelvis-fractured patient, the finite element model of the pelvis was reconstructed using the 3D medical image processing software Mimics (Materialise Inc., Belgium). It is worth mentioning that in the model reconstruction, we manually reunited the fractured parts to guarantee its mechanical stability, and then focused on the stress distribution caused by the pin insertion. Four identical external fixator pins made by stainless steel 305 with 5 mm in diameter and 124–175 mm in length were modeled using another commercial software Abaqus (Dassault Systèmes Simula, USA).

The pelvis was geometrically defined as a composite structure with a 3 mm thickened shell of cortical bone enclosing spongy bone [9]. Here, the sacrum was only considered to be consisted of cortical bone, as we mainly focused on the stress distribution of the fractured pelvis caused by different pin-inserting depths. All the materials were considered to be linear-elastic and isotropic, and their parameters were listed in Table 2. The shell element S3 was assigned to the cortical part in the pelvis, and the solid element C3D4 was assigned to the remaining structures including spongy bone in the ilium, sacrum, and the fixator pin. For the assembled model, the total element number in case of insertion 30 mm was 227,299 (C3D4: 178952, S3: 48347), and that in case of insertion 70 mm was 204,071 (C3D4: 162862, S3: 41209). In the simulation, the contact of cortical bone and spongy bone in the ilium was set to be coupled, and that of the sacrum and ilium was defined to be tied, which was also applied for the contact between the pins and the pelvis. The defined tie contact, constraining and loading conditions of the simulations were shown in Supplementary Figure S1.

**Table 2:** Material parameters of the model

Items	Young’s modulus (MPa)	Poisson’s ratio	Element type
Pelvis			
Cortical part	12,000 [10]	0.3 [10]	S3
Spongy part	100 [10]	0.2 [10]	C3D4
Sacrum	12,000 [10]^a^	0.3 [10]^a^	C3D4
Fixator pin	200,000 [11]	0.28 [11]	C3D4

^a^Because the sacrum was treated to be only consisted of cortical bone, its materials parameters were set as the cortical part in pelvis

We firstly performed a sensitivity analysis by increasing the element number in the simulation. Then, we considered two loading conditions, which shared a concentrated force of 500 N (a physiological loading of the body weight), [12, 13]. One condition occurred in the vertical direction, and the force was applied at the center of the top surface of the sacrum. The bottom of the pelvis was fixed by referring to a previous study where the acetabula were not taken into account neither [9]. The other was in the horizontal direction, and the force acted on the right peak point of the iliac crest while the left side of the iliac crest was fixed (see Supplementary Figure S1).

### Statistical analysis

All data were presented as mean ± standard deviation (SD) values. To evaluate whether the trajectories were precise, the parameters of external fixator pins including entry point, convergence angle and caudal angle were measured and compared before and after the operation by paired t-test. The *P* value was two-sided, and values less than 0.05 were considered statistically significant. All statistical analyses were carried out using SPSS 13.0 (SPSS Inc., Chicago, IL, USA).

## Results

### Preoperative measurement and simulation operation

**Fig. 2:**
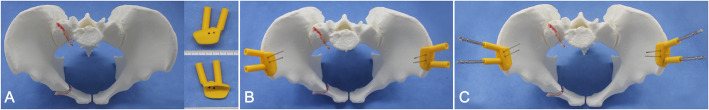
Simulated operation with the 3D-printed model and navigation template. **a** The pelvis model and the navigation template. **b** The navigation template was matched to verify the degree of surface feature matching. The Kirschner wire needle was placed to fix the template. **c** The external fixator pins were inserted according to the navigation hole

Using 3D reconstruction, the precise measurements of the pelvis were obtained. The entry point, direction of the pin, and the depth into the bone were successfully calculated using the software. Furthermore, the 3D printer accurately produced the pelvis model and navigation template using medical PLA (polylactic acid) material (Fig. 2a). The pin insertion channels in the template (Diameter: 6 mm; Length: 30 mm) were well matched to the external fixator pin (Diameter: 5 mm; Length: 150 mm). The insertion of pins was easily completed by the surgeon using the navigation templates. The evaluation of the pelvis model showed that the trajectories were consistent with the preoperative plans (Fig. 2b, c).

### Surgical outcome

**Fig. 3:**
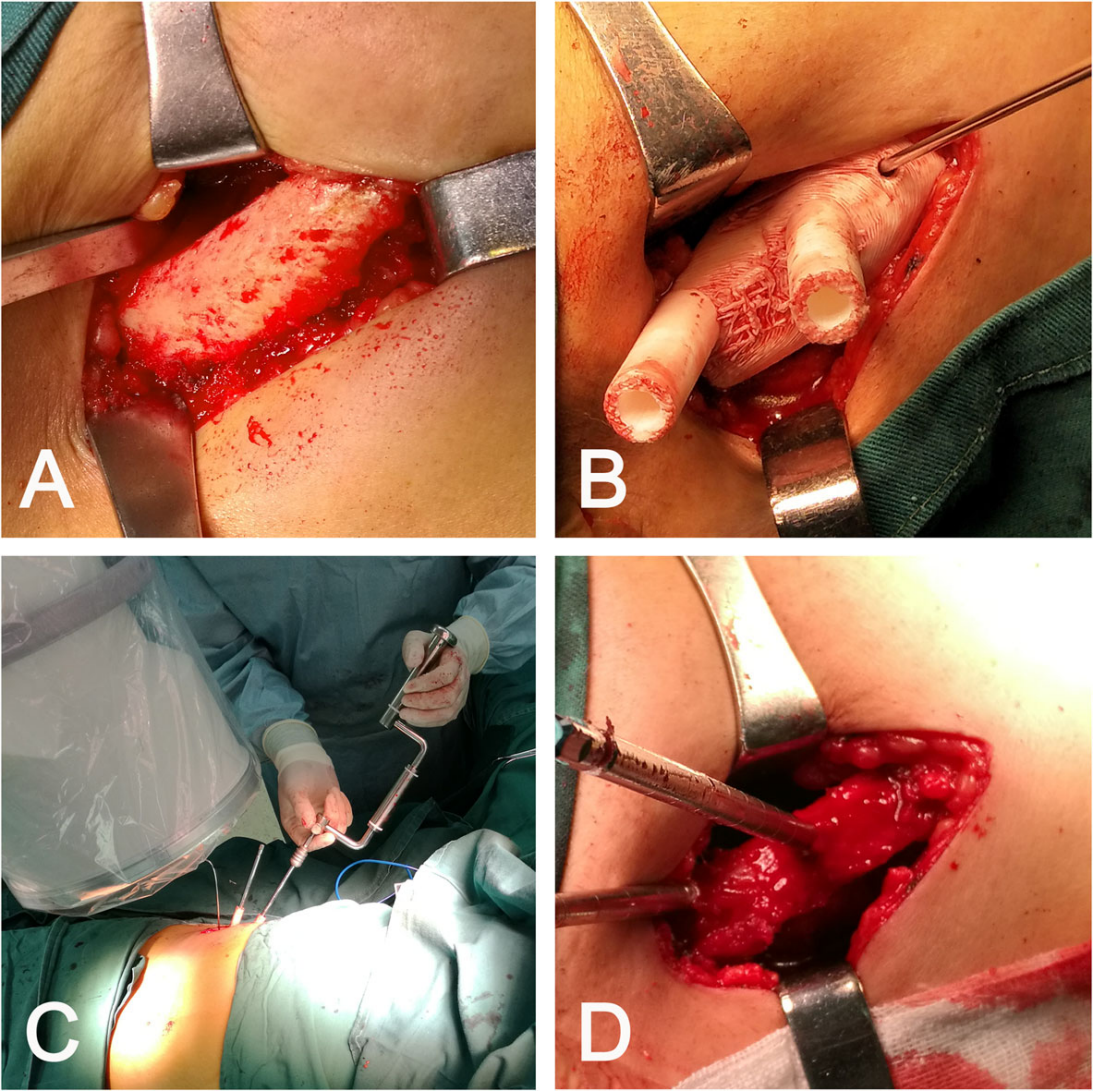
The navigation template applied in the operation on a pelvis fracture patient. **a** The soft tissue was carefully separated to reveal the base of the iliac crest and the inner plate of the ilium. **b** The navigation template was matched to the iliac crest. **c** The navigation template was used to guide the insertion of the pin. **d** The intraoperative process was completed as performed in the simulated operation

**Fig. 4:**
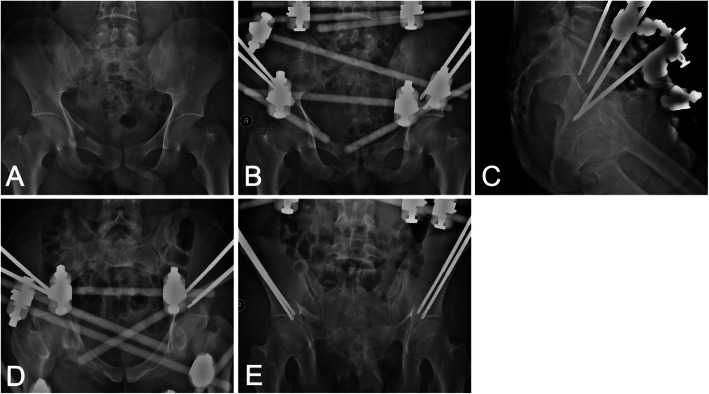
Preoperative and postoperative X-ray of the pelvis. **a** Preoperative X-ray, **b** Postoperative X-ray in the AP plane, **c** Postoperative X-ray in the lateral projection, **d** Postoperative X-ray in the inlet projection, **e** Postoperative X-ray in the outlet projection

**Fig. 5:**
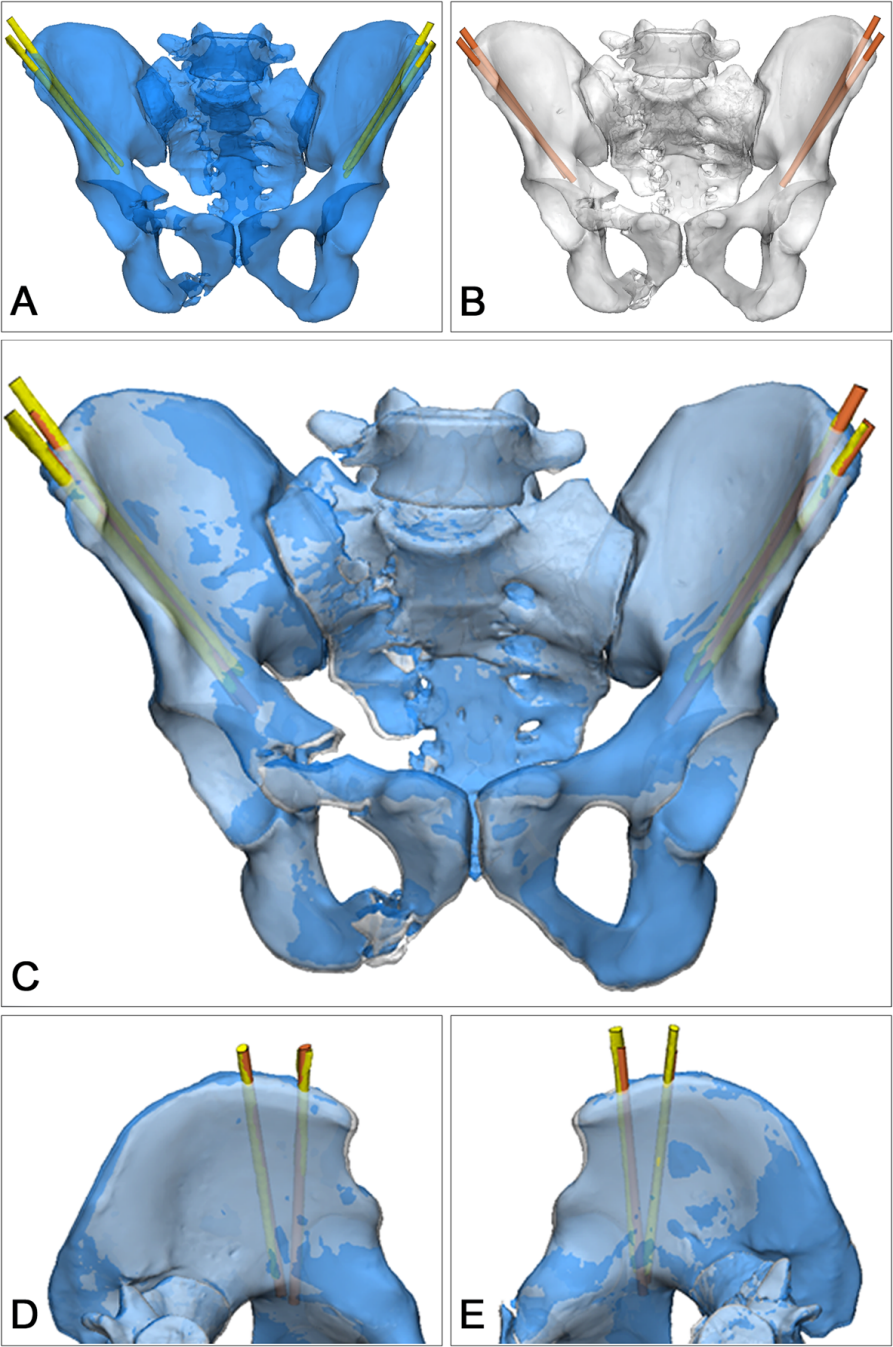
The overlay of trajectories before and after the operation. The trajectories after the operation (**a**) showed all pin was inserted pointing to the posterior column of the acetabulum, consistent with those in the preoperative plans (**b**). The merged images (**c**-**e**) confirmed the preoperative and postoperative trajectories were mostly overlaid by visual inspection

All external fixator pins were inserted using the individual drill template (Fig. 3). According to the postoperative CT scan, the average depth of all pins was 84.82 ± 10.48 mm. No pin penetrated the cortex of the ilium during surgery (Fig. 4). The postoperative CT scan of the pelvis was obtained using similar scan parameters. Original DICOM data were imported into Mimics 17.0 software. The trajectories were precise and consistent with the preoperative plans (Fig. 5). No significant difference in the entry point, convergence angle, and caudal angle of the pins were noted before and after the operation (all *P* > 0.05) (Table 3).

**Table 3:** Comparison of parameters before and after the surgery

Parameters	Before surgery	After surgery	*p*
*d* (mm)	41.07 ± 11.07	41.16 ± 10.97	0.883
A (^o^)	40.59 ± 7.83	40.89 ± 7.93	0.555
B (^o^)	36.10 ± 8.69	36.62 ± 9.12	0.510

*d*: The distance from the entry point to the anterior superior iliac spine. A: The convergence angle. B: The caudal angle

All eight patients were followed up for 3 months. No significant external fixation related complications occurred during the entire follow-up period, including loosening and rupture of pins, injury to blood vessels and nervous tissue, infection, and skin irritation. All patients were satisfied with the course and result of the treatment.

### The finite element model and mechanical analysis

According to the sensitivity analysis by increasing the element number (from 204,071 to 320,081) in the 30 mm model, we found a slight difference (less than 5%) in the maximum von Mises stress of the pelvis, which guaranteed the model accuracy.

**Fig. 6:**
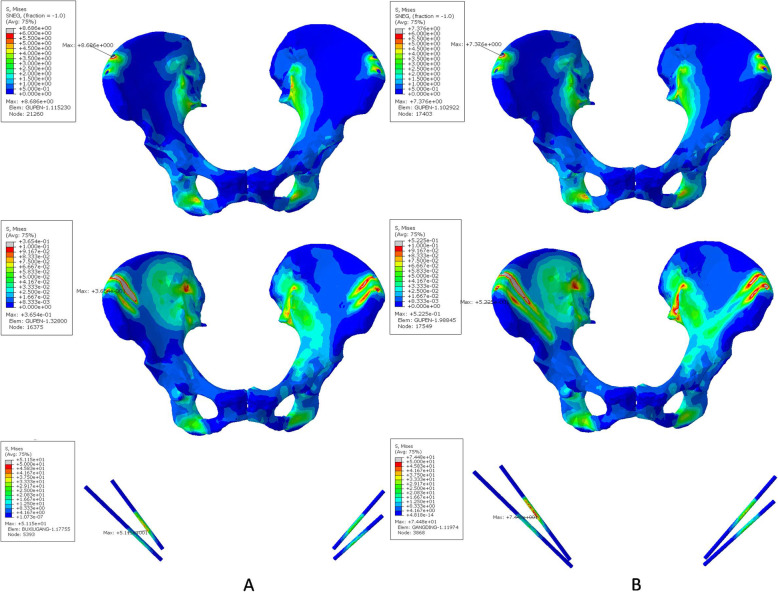
Von Mises stress distributions of the cortical, spongy bone and inserted pins in the pin-fixed pelvis-fractured model under vertical loading (Unit: MPa). **a**. Model with 30 mm pin insertion. **b**. Model with 70 mm pin insertion

The von Mises stresses of the cortical part, spongy part and pins under vertical loading showed the roughly symmetrical distribution in both cases (Fig. 6). For the 30 mm case (Fig. 6a), the maximum stresses of the cortical and spongy parts were at the right-side entry point of the pelvis, they were 17.950 MPa and 0.898 MPa, respectively. For the 70 mm case (Fig. 6b), the maximum stresses of the cortical and spongy parts were at the bottom (7.097 MPa) and the left-side entry point (1.895 MPa) of the pelvis. The maximum stress of pins in the 70 mm case was 68.220 MPa, which was greater than that 44.560 MPa of the 30 mm case.

**Fig. 7:**
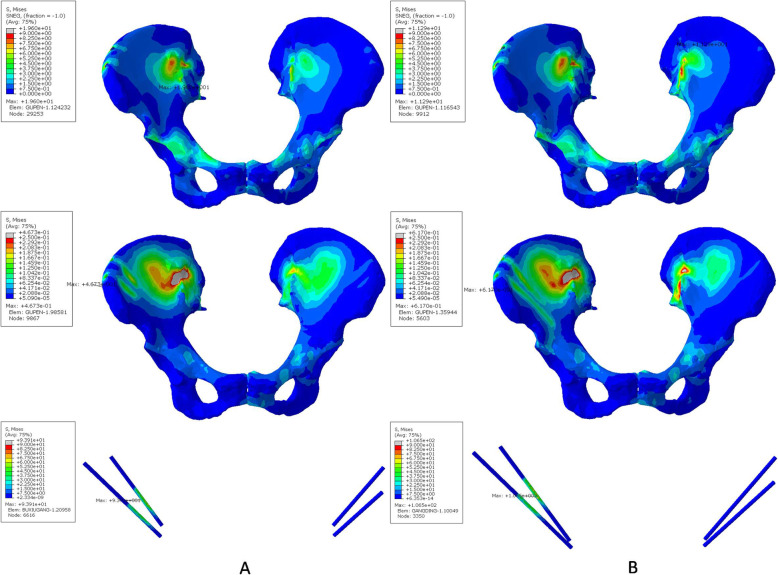
Von Mises stress distributions of the cortical, spongy bone and inserted pins in the pin-fixed pelvis-fractured model under horizontal loading (Unit: MPa). **a**. Model with 30 mm pin insertion. **b**. Model with 70 mm pin insertion

The von Mises stresses of the cortical part, spongy part and pins under horizontal loading resulted in an asymmetrical distribution (Fig. 7). For the 30 mm case (Fig. 7a), the maximum stresses of the cortical and spongy parts were at the right iliac fossa (19.690 MPa) and right-side entry point (1.037 MPa) of the pelvis. For the 70 mm case (Fig. 7b), the maximum stresses of the cortical and spongy parts were at the left iliac fossa (12.420 MPa) and right-side entry point (1.099 MPa) of the pelvis. Different from the vertical loading, the maximum stress of pins in the 70 mm case was 71.840 MPa, which was less than that 93.710 MPa of the 30 mm case.

## Discussion

In this study, all pins in simulation module was inserted from the iliac crest toward the supraacetabular region, and converged on the bone mass of the posterior column of the acetabulum. According to the postoperative CT scan, the pin insertion direction was well consistent with the preoperative plans. Furthermore, an individual navigation template was used as part of the surgical procedure for eight patients. Thirty-two external fixator pins were inserted using the individual navigation template. According to the postoperative CT scan, the average depth of the pins was 84.82 mm. No pin penetrated the cortex of the ilium during the operation.

No significant difference in the entry point, convergence angle, and caudal angle of the pins were noted before and after the operation, indicating that the trajectories were precise and consistent with the preoperative plans. These findings were different from that of the previous studies, and our results showed that the pin position matched with that of the computer design [14, 15]. The match may be attributed to the following: First, due to the regular and superficial surface of the iliac crest, it was easy for the navigation template to fit the surface of the iliac crest well. Second, the thin layer CT scan reduced the distortion of data conversion and 3D construction, which effectively controlled the error in data transformation and editing. Third, the design of the pin insertion channel was based on digital 3D construction, which could find a suitable path from multiple planes, and improve accuracy and repeatability during the operation. Fourth, during template designing, the identification mark in multiple planes improved the ability to fit the surface of the iliac crest, and long insertion channels in the template controlled the pin insertion position effectively. Finally, medical PLA material with certain hardness and toughness was selected for the template preparation, which proved to be convenient for preoperative disinfection and intraoperative application.

The primary treatment modality in case of a pelvic fracture focuses on controlling bleeding, restoring stability, and preventing complications. The instability of the pelvis fracture is the major reason for excessive bleeding. Bleeding is often difficult to control; therefore, the reduction and fixation need to be performed as soon as a pelvis fracture is diagnosed [16]. Balbachevsky et al. reported that external fixation is used for more than 79.5% of patients with a pelvic fracture, especially for patients with multiple injuries and soft tissue damage (1). However, clinical follow-up results showed that external fixation frequently causes infections and pin loosening (in more than 50% patients) and does not effectively stabilize the pelvis, indicating that the current method of external fixation needs to be improved clinically [17, 18].

In a previous study by Hiesterman et al., either a locking reconstruction plate or a spinal rod was placed through a subcutaneous tunnel, and the fixation into the iliac crest and pubis was achieved to effect stability. At 6-month follow-up, there was only one pin tract infection out of all the 11 patients [19]. Noda et al. reported that the biplanar external fixation could provide better stability than the uniplanar external fixation and minimize pin-site infection [20]. Archdeacon et al. compared the structural stiffness of a pin construct with 2 models of parallel pin constructs and found a significantly stiffer construct for in-plane loading compared with either parallel pin construct [21]. Meanwhile, a few reports have supported that external fixation on supra-acetabulum has a good stiff construct because the bone condition of supra-acetabulum is beneficial for the deep pin position. Therefore, there are several limitations of external fixation on supra-acetabulum. First, it is difficult to expose the anterior inferior iliac spine, identify the entry point, and control pin insertion position [22]. Second, the pin insertion requires splitting of the sartorius and iliac muscles, which may cause an injury to the lateral femoral cutaneous nerve and irritates the deep soft tissue [23]. Third, although the tip of the pin in the supra-acetabulum could reach the sacroiliac joint, the average depth of the pin position using the standard method was approximately 50 mm [6], and the insertion channel is surrounded by cancellous bone resulting in a broad fixed area, which influences the mechanical properties of the external fixation.

The stability and pin-related complications following external fixation is closely related to the pin insertion position [6, 7, 24]. Gardner et al. reported that the external fixation could achieve compression and stabilization of the sacroiliac joint when the pins were placed completely to the posterior superior iliac spine [6]. Ponsen et al. reported that external fixator of pelvic ring could improve stability for the treatment of type C pelvic fracture by an increase in pin diameter and alternative pin position [7]. The high rate of infection and pin-related loosening was caused by the poor direction and shallow depth of the external fixator pin using the traditional external fixation on the iliac crest [24]. In the present study, the pin insertion procedure was modified from that of the traditional method. All pins were inserted into the supraacetabular region, where the highest bone density in the human body could be found [6]. The individual template could improve the precision of the external fixator pin position, and increase the depth compared with the traditional method (50 mm) [4]. No pin-related complication such as infection and loosening were found during the postoperative follow-up. Finite element analysis showed the deeper pin position (70 mm in our study) reduced the maximum stress of the cortical part but slightly increased the counterpart of the spongy part. This indicated that the deeper pin position could rationalize the maximum von Mises stresses of the pelvis.

There are several limitations to our current study. First, compared with percutaneous approach in the traditional method, our method requires stripping of the soft tissue on the iliac crest and the inner plate of the ilium for the template attaching, which cause about 4 cm skin incision and local soft tissue invasions. Moreover, it is noted that such method may not be suitable for emergency surgeries as it will take about 4 h for the preparation and 2 h for sterilization of individual templates before operation. Second, the number of patients was relatively small, more patients are warranted to confirm the reproducibility of our study. The mechanical difference among several external fixation methods should be analyzed to strengthen the statistic power in the future study. Moreover, strictly validating the model in the current study was difficult because cadaveric specimens from available donors was absent. A high credibility of the model should be provided by in vitro experiments with cadavers for potential clinical use in the future [25].

## Conclusions

The individual navigation template is an improvement of the traditional method. The method could increase precision and depth of pin position. The deeper pin position could rationalize the maximum von Mises stresses of the cortical and spongy bones of the pelvis. Finally, the approach resulted in good mechanical stability and low risk of pin-related complications.

## Supplementary information

**Additional file 1: Figure S1.** The defined tie contact, constraining and loading conditions in the simulations. (A) Definition of the tie contact was shown in the simulations. (B) One condition occurred in the vertical direction, and the force (i.e., RP-1) was applied at the center of the top surface of the sacrum. To be consistent with the surgical procedure, points a, b, c, d were defined as the locations of the connectors, and the distances between points a, b, c, d and entry points of the pelvis were uniformly set as 30 mm. The four points were coupled with the defined four reference pins (RP3-RP6) on which the constraining conditions were applied. (C) The other condition was in the horizontal direction, and the force (i.e., RP-2) acted on the right peak point of the iliac crest while the left side of the iliac crest was fixed.

## Data Availability

The datasets analyzed during the current study are available from the corresponding author on reasonable request.

## Abbreviations

RP: Rapid prototyping
PLA: polylactic acid
