# Supplementary figures and images for: A feasibility study of individual 3D-printed navigation template for the deep external fixator pin position on the iliac crest
Source: BMC Musculoskelet Disord. 2020 Jul 21;21:478. doi: 10.1186/s12891-020-03509-6 (PMC7372844; doi:10.1186/s12891-020-03509-6)

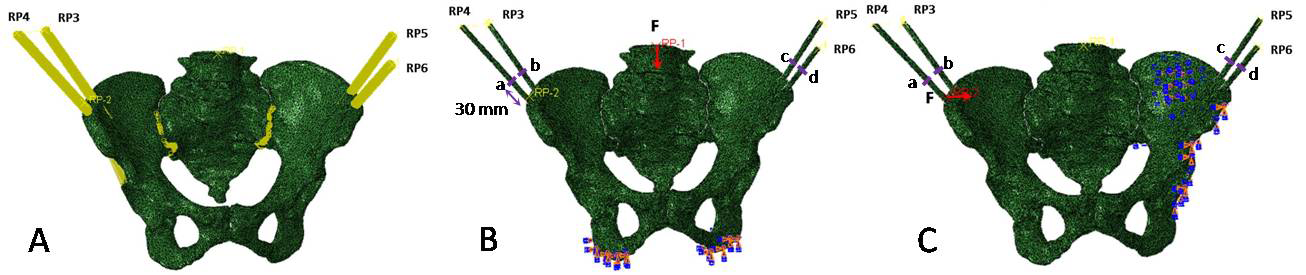

Supplement: Supplementary file 1 — Additional file 1: Figure S1. The defined tie contact, constraining and loading conditions in the simulations. (A) Definition of the tie contact was shown in the simulations. (B) One condition occurred in the vertical direction, and the force (i.e., RP-1) was applied at the center of the top surface of the sacrum. To be consistent with the surgical procedure, points a, b, c, d were defined as the locations of the connectors, and the distances between points a, b, c, d and entry points of the pelvis were uniformly set as 30 mm. The four points were coupled with the defined four reference pins (RP3-RP6) on which the constraining conditions were applied. (C) The other condition was in the horizontal direction, and the force (i.e., RP-2) acted on the right peak point of the iliac crest while the left side of the iliac crest was fixed. [file 12891_2020_3509_MOESM1_ESM.tif]
